# Supplementary material for: Environmental and Circadian Regulation Combine to Shape the Rhythmic Selenoproteome
Source: Cells. 2022 Jan 20;11(3):340. doi: 10.3390/cells11030340 (PMC8834552; doi:10.3390/cells11030340)
Supplement: Supplementary file 1 [file cells-11-00340-s001.zip › Supp Figures.pdf]

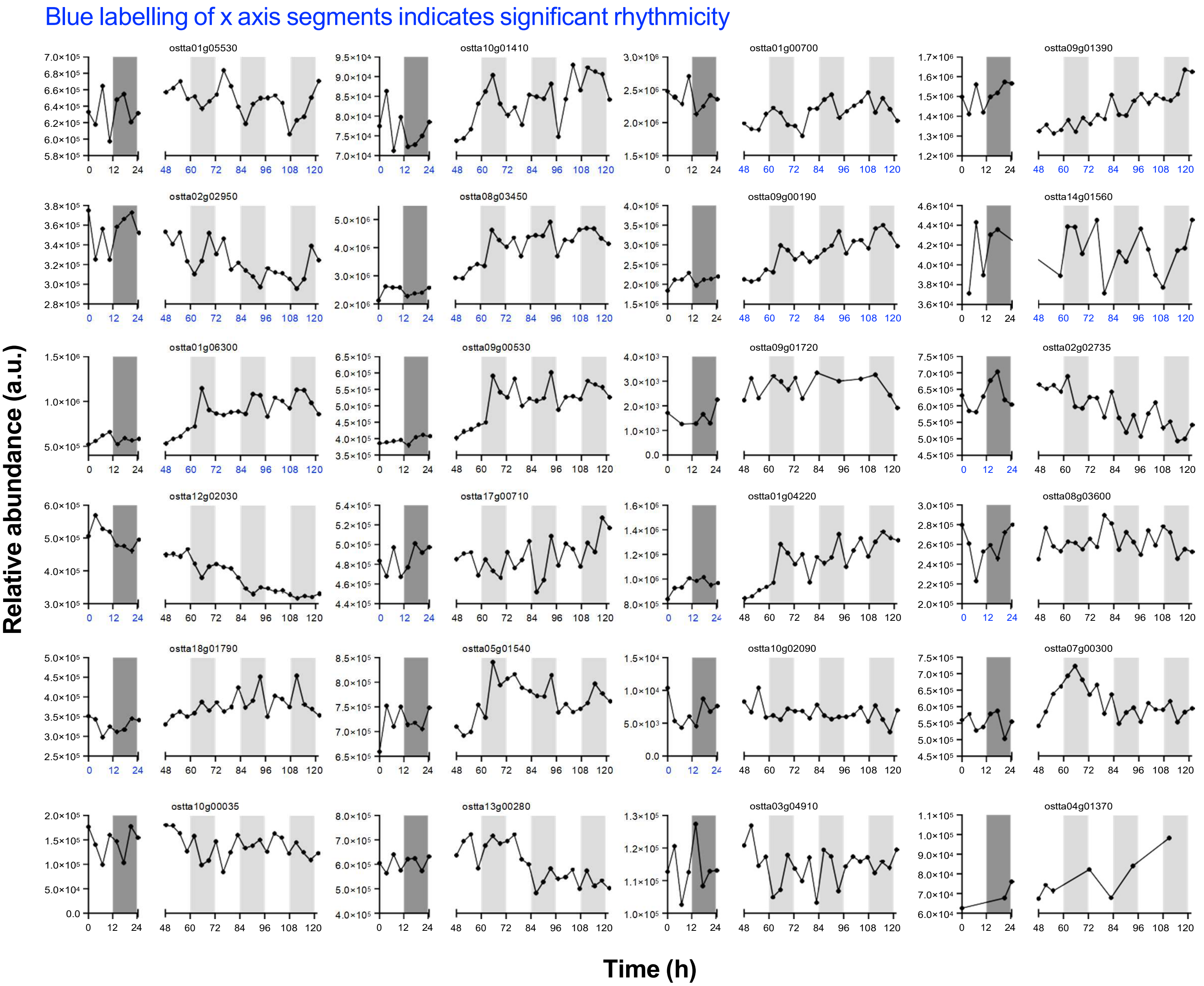

Supplemental Figure S1: Abundance profiles of all selenoproteins under LD and LL conditions. Abundance profiles of all 24 selenoproteins over a single cycle of light-dark (0-24h) and three cycles of constant light (48-120h). Proteins are ordered by p-value for rhythmicity under LL. If a protein is significantly rhythmic in LL, this is reflected by blue numbering of the time axis for LL timepoints. If a protein is significantly rhythmic under LD, this is reflected by blue numbering of the LD time axis. Three proteins are rhyhtmic under both conditions.

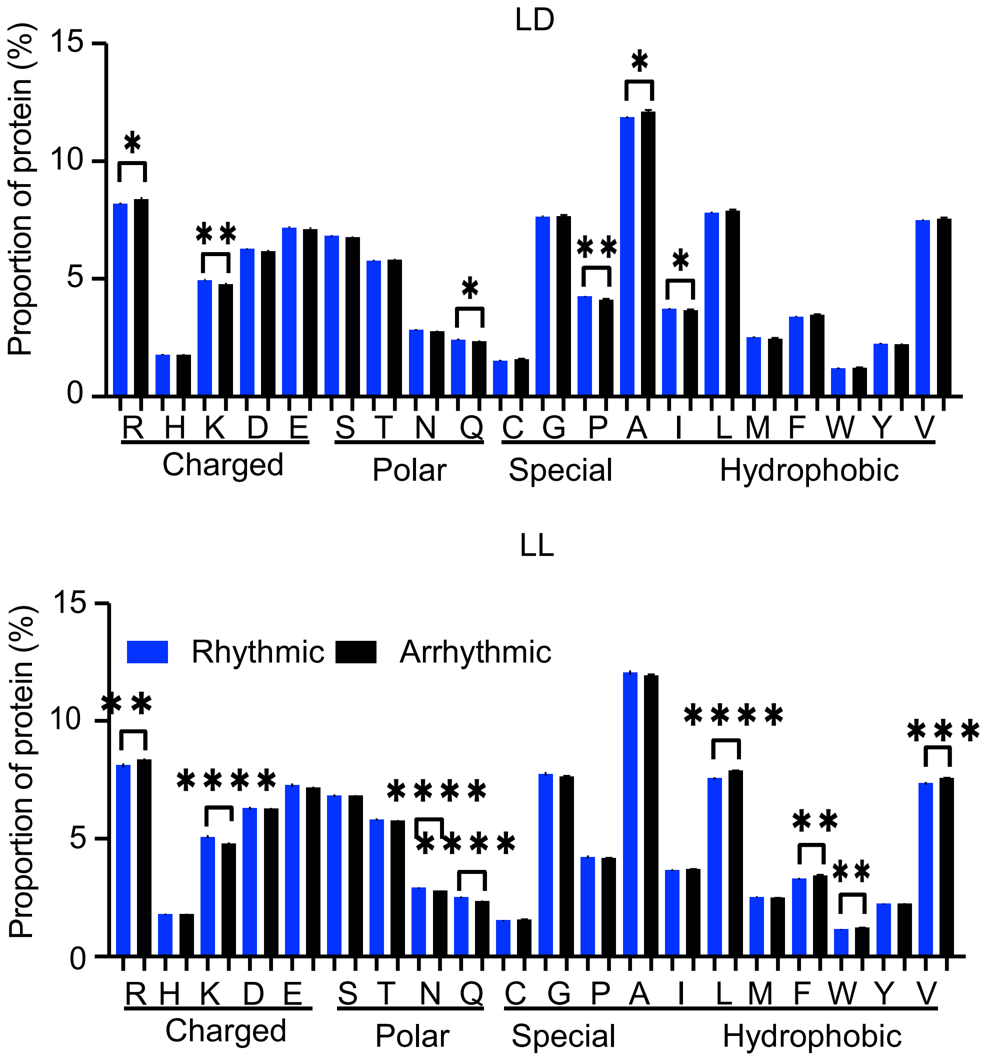

Supplemental Figure S2: Amino acid usage among rhythmic versus arrhythmic proteins

Amino acid usage for the 20 directly encoded proteinogenic amino acids, expressed as the average proportion of a protein amongst rhythmic versus arrhythmic proteins in LD (top) or LL (bottom). Statistics are unpaired nonparametric tests (Mann-Whitney); **** p value <0.0001; *** p value <0.001; **p value <0.01; * p value <0.05.
